# Supplementary material for: WayFindR: investigating feedback in biological pathways
Source: NAR Genom Bioinform. 2026 May 19;8(2):lqag051. doi: 10.1093/nargab/lqag051 (PMC13183675; doi:10.1093/nargab/lqag051)
Supplement: lqag051_Supplemental_File [file lqag051_supplemental_file.docx]

**Supplementary Materials**

**KEGG**

We extracted 328 human pathways from the KEGG database and systematically analyzed them for the presence of feedback loops. Among these, 91 pathways contained at least one cyclic structure. Of those, only 10 pathways featured cycles with at least one inhibitory edge. In total, we identified 23 negative feedback loops across these 10 pathways.

The lengths of negative feedback loops ranged from 4 to 12 nodes (**Figure 6**). The most common node types within these loops were “gene” and “map,” with “activation/phosphorylation” being the predominant edge subtypes connecting them.


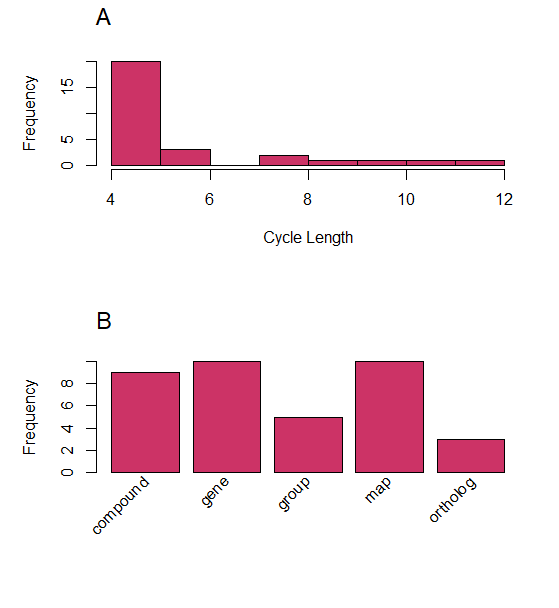


**Figure 6. (A)** Distribution of negative feedback loop lengths; **(B)** Frequency of node types involved in negative feedback loops across KEGG human pathways.

The distribution of subtype pair combinations in **Table 5** reveals several notable patterns. Activation-related interactions are the most prevalent, particularly activation/phosphorylation, which occurs far more frequently than any other combination. This suggests that phosphorylation-mediated activation plays a central role in negative feedback regulation within KEGG human pathways. In contrast, inhibitory interactions such as inhibition/dephosphorylation and inhibition/phosphorylation are also well represented, indicating that both activation and inhibition mechanisms are tightly coordinated in feedback control.

Less frequent combinations, including methylation- and ubiquitination-related interactions, appear only rarely, suggesting that these mechanisms may play more specialized or context-dependent roles. Overall, the observed distribution highlights a dominance of phosphorylation-driven regulatory processes, with indirect effects and inhibitory mechanisms contributing to the fine-tuning of negative feedback loops.

| **Subtype Combination** | **Frequency** |
| --- | --- |
| activation/phosphorylation | 86 |
| activation/indirect effect | 36 |
| inhibition/dephosphorylation | 34 |
| inhibition/phosphorylation | 27 |
| inhibition/indirect effect | 11 |
| expression/indirect effect | 11 |
| activation/binding/association | 2 |
| activation/methylation | 1 |
| inhibition/methylation | 1 |
| activation/ubiquitination | 1 |

**Table 5.** Subtype pair frequencies in KEGG human pathways exhibiting negative feedback.

Moreover, we checked how many times each gene appeared across all 23 feedback loops. This revealed that 97 unique genes are involved, with CISH and STAT3 being the most frequently recurring, each appearing 10 times. This high recurrence aligns with their known roles in cytokine signaling pathways. Specifically, STAT3 is activated by cytokines and translocates to the nucleus to induce target genes, including CISH. In turn, CISH acts as a feedback inhibitor by suppressing further STAT signaling.

Functional enrichment analysis of the 97 genes participating in negative feedback loops revealed a significant overrepresentation of cellular and hormonal response processes (**Figure 7**). The most enriched categories included cellular response to peptide and response to peptide hormone. Additionally, enrichment in regulation of trans-synaptic signaling and peptidyl-serine modification indicates a role in neuronal communication and post-translational modifications, both of which are critical for fine-tuning dynamic responses in complex signaling systems.


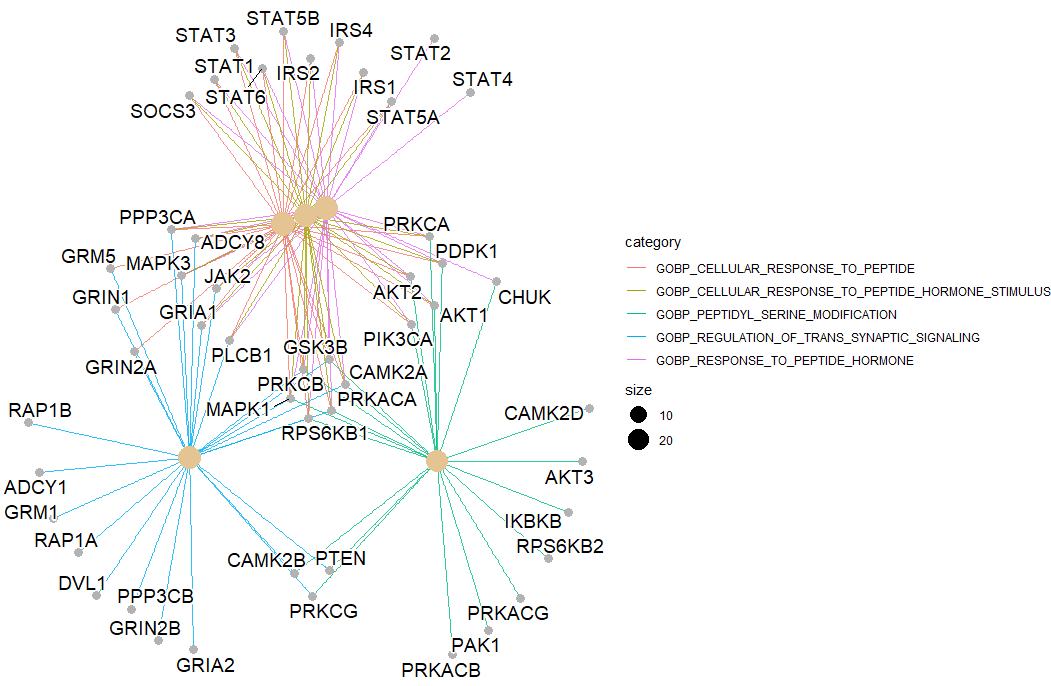


**Figure 7.** The linkages of overrepresented genes in negative feedback loops extracted from KEGG human pathways and biological concepts (GO terms) as a network.
